# Mutational Patterns Cannot Explain Genome Composition: Are There Any Neutral Sites in the Genomes of Bacteria?

**DOI:** 10.1371/journal.pgen.1001104

**Published:** 2010-09-09

**Authors:** Eduardo P. C. Rocha, Edward J. Feil

**Affiliations:** 1 Institut Pasteur, Microbial Evolutionary Genomics, Département Génomes et Génétique, Paris, France; 2 CNRS, URA2171, Paris, France; 3 Department of Biology and Biochemistry, University of Bath, Claverton Down, Bath, United Kingdom; University of Arizona, United States of America

**Figure 1:**
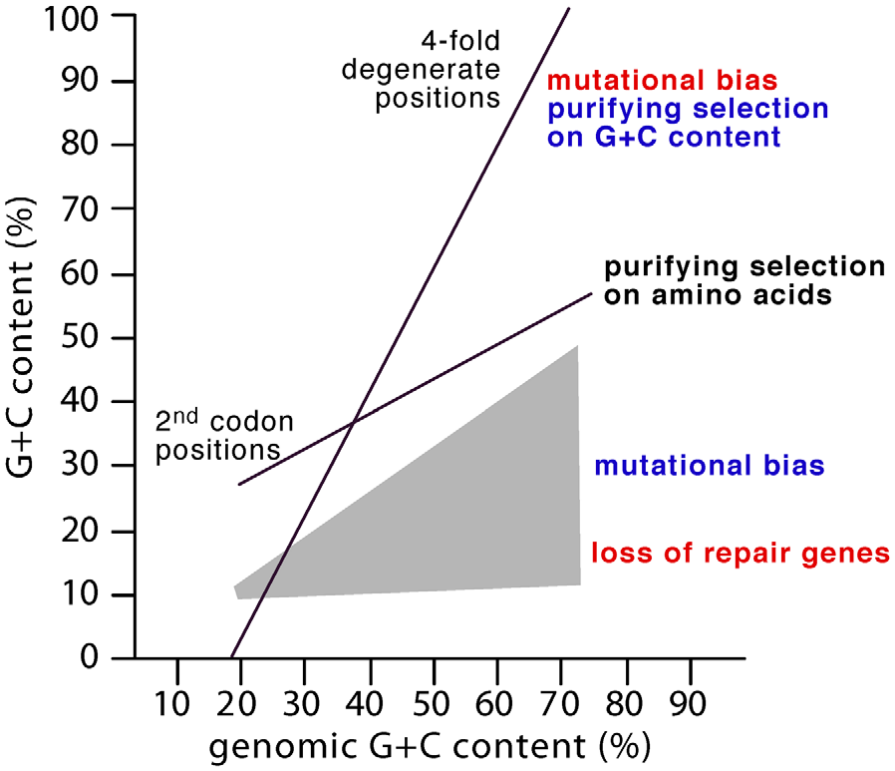
The GC composition of genomes is strongly correlated with second codon (GC_2_) and 4-fold degenerate positions (GC_4_) [**2**]. Second codon positions show low variability due to purifying selection on non-synonymous changes. 4-fold degenerate positions vary between 5% and 97% GC among published genomes. In the classical neutral scenario (red), 4-fold degenerate positions are nearly neutral and their composition results essentially from mutational patterns. These patterns are modified in bacteria that lose repair genes, such as mutators, which show additional AT pressure (grey area) [19]. In the selectionist view (blue), the composition of 4-fold degenerate positions results from selection for GC content, the mutational patterns are AT-rich relative to genome composition, and there are no neutral positions. Naturally, this is an idealized view of genomes that code for many additional overlapping signals that are under selection, e.g., codon usage bias, regulatory signals, etc.

The dissection of natural selection and neutral processes remains a core problem for molecular evolutionary biologists. One of the longest-standing controversies concerns the causes of genome base composition, notably the variation in the sum of G and C content (GC) between 17% and 75% in bacteria. Sueoka argued very early that GC content variation is driven by mutational biases and, as this bias affects non-synonymous sites, protein evolution might also be largely driven by neutral forces [1]. Later, Muto and Osawa showed that 4-fold degenerate positions in codons exhibit the largest range of GC content (GC_4_), whereas the non-degenerate second codon positions (GC_2_) exhibit the narrowest (Figure 1) [2]. As the footprint of genomic GC variation is most evident in those sites under the least selective constraint for amino acid composition, it has become accepted that GC content variation is primarily driven by neutral mutational effects and has little adaptive relevance [2].

Two papers in the current issue of *PLoS Genetics* aim to test whether the variation in bacterial genomic GC content results directly from mutation biases. Far from observing variation in mutational patterns concordant with the range of GC content, Hildebrand et al. [3], and Hershberg and Petrov [4] independently point to a strong and consistent AT pressure on bacterial genomes, whereby *de novo* GC → AT mutations arise much more commonly than the reverse. Hershberg predicts that most bacterial genomes, if left entirely vulnerable to mutation, would approach an equilibrium GC content of 20%–30%, close to the highly reduced genomes of endosymbionts [5]. Discounting a rather implausible scenario whereby nearly all diverse GC-rich taxa are converging towards a low GC content, one is forced to conclude that the excess A and T generated by mutation bias (AT pressure) is lost over time. If so, mutational patterns are not strongly shaping genomes after all, and something else is keeping GC contents up.

Hildebrand and co-workers analyze polymorphism data from 149 phylogenetically diverse species corresponding to a wide range of GC content. A major strength of this analysis is that it tests for a number of possible confounders that might explain the excess of GC → AT changes, including variation in mutation rates, sequencing errors, and violations of the infinite sites assumption. The proportion of GC ↔ AT changes that are GC → AT (Z) is almost always >0.5, and is positively correlated with GC_4_. This means that AT pressure is strongest in GC-rich genomes. For the most GC-poor genomes, the ratio is reversed (Z<0.5), but this might result from violation of the infinite sites assumption at extreme GC content. In fact, the extreme AT-rich genomes of *Buchnera* do have Z = 0.5 [6].

Hershberg and Petrov exploit full genome data of five very recently evolved “clonal pathogens”, presumably under relaxed selection, allowing precise detection of mutational patterns. This more limited dataset includes no extreme GC-poor genomes. On the other hand, the availability of a large number of SNPs and of an outgroup allows the comparison of patterns within and between species. Consistent with the results of Hildebrand et al., Hershberg and Petrov find an excess of GC → AT mutations in synonymous, non-synonymous, and intergenic sites. Comparisons with the outgroup species suggest this is not caused by loss of repair genes, and that it abates over greater phylogenetic distances (i.e., between “species”). This pattern is similar to that previously found in *E. coli* [7], and reflects the action of purifying selection (or a process that mimics selection) preferentially removing AT-enriching mutations over time. Hershberg and Petrov's study also highlights the significance of weaker purifying selection in newly emerged pathogens, as shown in *Shigella* strains [7]. Strikingly, they find no evidence for a correlation between predicted GC contents at mutational equilibrium and extant base composition, suggesting that mutational bias might have no role in shaping genome composition. Hildebrand et al. show a similar qualitative bias, but predicted equilibrium values vary between 5% and 90% GC. As methods and datasets differ in the two studies, further analyses will be required to shed light on this issue.

Taken together, the evidence for a common mutational pressure towards low GC is clear. The process maintaining base composition in GC-rich genomes must be very strong, because a genomic GC content of 75% corresponds to a GC_4_ of nearly 100% (Figure 1). This represents a ∼70% gap with Hershberg and Petrov's predicted mutational equilibrium. Two distinct processes might be at work: biased gene conversion (BGC) and natural selection.

In certain eukaryotes, BGC results from recombination between heterologous sequences preferentially removing AT polymorphisms [8]. Contrary to sexual eukaryotes, allelic recombination in bacteria requires horizontal transfer. As a result, rates of recombination between, and even within, different bacterial species are notoriously variable. Consistent with the action of BGC, ecologically isolated endosymbionts do not recombine and have extremely rich AT genomes [5], and regions of high recombination in *E. coli* are also GC rich [9]. Yet, Hildebrand et al. found qualitatively similar results when excluding taxa with evidence for recombination. Hershberg and Petrov mostly use nearly clonal genomes and still find a large gap between mutation patterns and genome composition. While available evidence suggests a weak role for BGC in the variation of GC content in bacteria, it is very difficult to completely rule out a role for BGC because it purges AT polymorphisms just like natural selection. As a result, recently emerged pathogens with an excess of AT polymorphisms experience both weakened selection and decreased recombination, both of which could potentially explain a decrease in GC content. More research is needed on the impact of BGC in bacterial genomes.

The alternative to BGC is that high GC contents are selectively maintained. Many explanations for GC content variation have been proposed (summarized in Table 1). GC content variation is most marked at synonymous and intergenic sites. Hence, any selective explanation for this variation forces us to turn the traditional concept of the “neutral site” on its head (Figure 1). In this new view, no single position is evolving neutrally in genomes. As a result, 4-fold degenerate positions are not the closest proxy to mutational patterns, but the result of selection for genomic GC content. If so, we are facing a seismic shift of paradigm in molecular evolution. Detection of adaptive features such as codon bias or amino acid frequencies currently rely on a background null hypothesis assumed to reflect neutrality. Neutral models are also the basis of coalescent-based studies of bacterial demography. If there are no neutral positions, then there is no neutral null by which to detect adaptation and we are required to first superimpose selection leading to genome composition in evolutionary studies.

**Table 1:** Variables Historically Proposed to Explain GC Variation in Prokaryotes.

Variable	Why?	But…
Background selection	GC-rich regions recombine more in *E. coli* [9], favoring background selection [20].	Unclear if the GC effect in recombination is general and strong enough to explain the observations.
Biased gene conversion	Repair resulting from conversion between mismatched sequences distorts sequence composition, increasing GC [8]. High recombination regions in *E. coli* are GC richer [9].	Recombination increases the efficiency of selection, and thus also facilitates selection for GC. BGC cannot explain GC richness in nearly clonal bacteria. Observed recombination/mutation ratios do not correlate with GC content [3].
DNA folding	In dsDNA, GC increases stability, whereas AT increasesflexibility [21].	Unclear if GC-based stability is selected for in dsDNA given the observed low effect of temperature on GC content and the preference for AT-rich sequences at promoters.
Environment	Different environments contain bacteria differently enriched in GC [13].	Mechanisms underlying this variable are unclear and could result from combinations of the other variables [14].
Gene length	GC richness favors large genes by reducing the frequency of non-sense mutations. Gene GC content correlates with its length [22].	Genomic GC content is at best weakly correlated with the average gene length, which does not vary widely between genomes [22].
Genome length	Genome reduction is often driven by low effective population size(Ne) [23]. Small genomes are GC poor and large genomes GC rich [12].	Gene density being high in prokaryotes, genome length is a proxy of many variables. This renders clear biological interpretations difficult.
Mutation pressure	Mutations are AT rich [3], [4], and loss of repair genes leads to AT enrichment [19].	Does not explain the compositional gap between mutation patterns and actual composition of genomes. Does not explain the existence of GC-rich genomes.
Nitrogen-fixation	Selection to save nitrogen (N) use in DNA and RNA because both are N-rich molecules, A/T/U having 7 and G/C 8 N atoms. GC content is higher in N-fixers [24].	GC content is higher in 2 genera of aerobic nitrogen fixers but lower in 2 anaerobic genera [24]. Most prokaryotes are not N-fixers.
Oxygen	Tightly packed GC-rich DNA might be less prone to oxidation. Synonymous Gs could have a sacrificial role in oxidizing environments. Aerobes are GC rich [11].	It's hard to envisage selection of GC polymorphisms for future sacrificial roles. In general, G is the nucleotide most prone to oxidation.
Parasitism	Pathogens, plasmids, transposable elements, and bacteriophages are enriched in the costless and abundant AT [10].	Does not explain the existence of GC-rich genomes.
Protein composition and folding	GC-rich codons encode amino acids biosynthetically cheaper[25]. Susceptibility to oxidation [26] and folding stability co-vary with GC [27].	Selection on GC should not be driven by protein composition because purifying selection on GC content is strongest at degenerate and intergenic sites.
RNA folding	Practically all positions in bacterial genomes are transcribed, and GC-rich RNA structures are more stable.	Only stable RNAs, not all mRNAs, are strongly enriched in GC in thermophiles [28]. Core genes have fairly homogeneous GC, and exceptions concern large genomic regions, not highly expressed operons [16]. rDNA operons, the most transcribed under exponential growth, are GC richer in AT-rich genomes and GC poorer in GC-rich genomes.
Speciation & self- recognition	Different GC contents would favor speciation and recognition of self- from non-self DNA [29].	It does not explain why there are traces of pervasive selection only for GC.
Temperature	GC richness increases thermostability of dsDNA, RNA structures, and codon-anticodon pairing [30].	Association of optimal growth temperature with genomic GC is weak at best [28], [31]. *Pasteurella* strains evolved at high temperatures became AT richer [32].
UV radiation	AT-rich dinucleotides are more susceptible to form pyrimidine dimmers upon UV irradiation [33].	No observable counter-selection of UV-susceptible dinucleotides [34].

Previous selective explanations for GC content variation are wide-ranging and include considerations of the cost and availability of nucleotides [10], aerobiosis [11], and genome length [12] (Table 1). Metagenomics analyses indicate a strong environmental component to GC content variation [13], [14], and it is intriguing that the most GC-rich taxa yet sequenced have very large genomes and live in the soil. Any selective explanation for GC content must tackle the problem of small selection coefficients at individual sites. This has been a long-standing argument against selection for temperature adaptation shaping mammalian isochores [8], [15]. However, bacteria have smaller genomes and supposedly much larger effective population sizes than mammals. This might facilitate the selection of mild-effect polymorphisms [16].

Even if one discovers a source of selection for GC content, basic questions will remain. For example, does GC variation reflect differences in the selective optima or just differences in the strength of selection? These and previous studies suggest that adoption of intimate associations with eukaryotes leads to a reduction in the effective population size and to AT enrichment, possibly due to less efficient purging of GC → AT mutations (but see [17]). But does it follow that GC-rich genomes are universally desirable, yet only achievable for taxa with a very large effective population size? Alternatively, intermediate GC contents might sometimes be optimal, e.g., because of trade-offs between traits associated with different explanatory variables. In this latter view, GC content variation would emerge through a combination of variation in selective optima and effective population sizes. One further intriguing question is, why haven't mutational patterns evolved towards generating the optimal composition in genomes? If it is confirmed that selection and mutation biases are always antagonistic in GC-rich genomes, what does this reveal about the mutation process?

Finally, are such biases peculiar to bacteria? In *Arabidopsis thaliana*, mutational patterns are also AT rich [18], and in mammals and birds there is evidence linking recombination rates with the rise in frequency of GC polymorphisms and isochore structure [8]. Could all such patterns be universally linked to the same biological processes? The ever-expanding sequencing output should soon allow extensive comparative studies to shed a great deal of light on these mysteries.
